# Rhinocerebral Mucormycosis With Brain Abscess Presenting as Status Epileptucus in a COVID-19-Infected Male: A Calamitous Complication

**DOI:** 10.7759/cureus.21061

**Published:** 2022-01-09

**Authors:** Vinay Verma, Sourya Acharya, Sunil Kumar, Shilpa A Gaidhane, Utkarsh Thatere

**Affiliations:** 1 Department of Medicine, Jawaharlal Nehru Medical College, Datta Meghe Institute of Medical Sciences (Deemed to be University), Wardha, IND; 2 School of Epidemiology and Public Health, Jawaharlal Nehru Medical College, Datta Meghe Institute of Medical Sciences (Deemed to be University), Wardha, IND

**Keywords:** covid 19, fess, seizure, status epilepticus, cerebral mucormycosis, brain abscess

## Abstract

With the evolution of COVID-19 disease, the emergence of more complications associated with COVID-19 is taking place. Mucormycosis is the most common opportunistic fungal infection encountered after COVID-19. In this case report, we describe a case of a 61-year-old male type 2 diabetic with sino-orbital-cerebral mucormycosis infection who was treated with conventional guidelines for a recent COVID-19 infection and further presented with generalized tonic-clonic status seizures. Neuroimaging revealed sino-orbital mucormycosis with right frontal lobe abscess. He was treated with anti-epileptics, steroids, amphotericin-B, and functional endoscopic sinus surgery (FESS).

## Introduction

The COVID-19 outbreak has rapidly spread on a worldwide scale [1,2]. There has not been a successful "cure" for the disease since the outbreak. Many drug therapies have been tried and tested but prevention, prophylaxis and symptomatic treatment have been deemed as the mainstay of management in these patients. Other respiratory viruses like influenza, SARS, MERS have a common phenomenon of secondary infections which have been reported in hospitalized and critically ill patients in about 10 to 30% cases, with the most common etiology being fungal (about 10 times more common) [3]. Mucormycosis caused by Mucorales species of the Zygomycota is a very lethal infection mostly affecting immunocompromised individuals like diabetics, people with leukemia or lymphoma [4]. The incidence of mucormycosis in India is about 0.14 per 1000 (about 80x developed countries) [5]. However, factors like intracranial or orbital involvement, irreversible immune suppression increases mortality to about 50-80% [6]. Immunocompromised patients should be anticipated for this disease.

India has contributed to about 70% of all Mucormycosis cases in post-COVID-19 patients [4]. Transmission of Mucormycosis occurs through inhalation, inoculation, or ingestion of spores from the environment. It most commonly affects the sinuses or the lungs after inhaling fungal spores from the air. In such cases, it may spread to the brain and eyes.

A hallmark of mucormycosis, tissue necrosis, is a late sign. The diagnosis of mucormycosis is difficult which often leads to a poor prognosis. This demonstrated the need for early diagnosis and management. If the treatment or diagnosis is delayed by seven days, the 30-day mortality is two times as compared to early initiation of treatment (35% to 66%). Despite early diagnosis and combination therapy (medical and surgical), there is a poor prognosis in patients with mucormycosis [5].

## Case presentation

A 61-year-old male, a resident of Wardha, presented to a primary health center with a generalized tonic-clonic seizure for 20 minutes. He was given initial treatment and was referred to a higher center and was then received in the emergency department of our hospital in a postictal state. His relatives gave a history of one episode of involuntary movements of hands and legs which lasted for 20 minutes and relived by itself 5 hours back. There was a history of frothing from the mouth, tongue bite, urinary incontinence and amnesia for the event.

**Figure 1:**
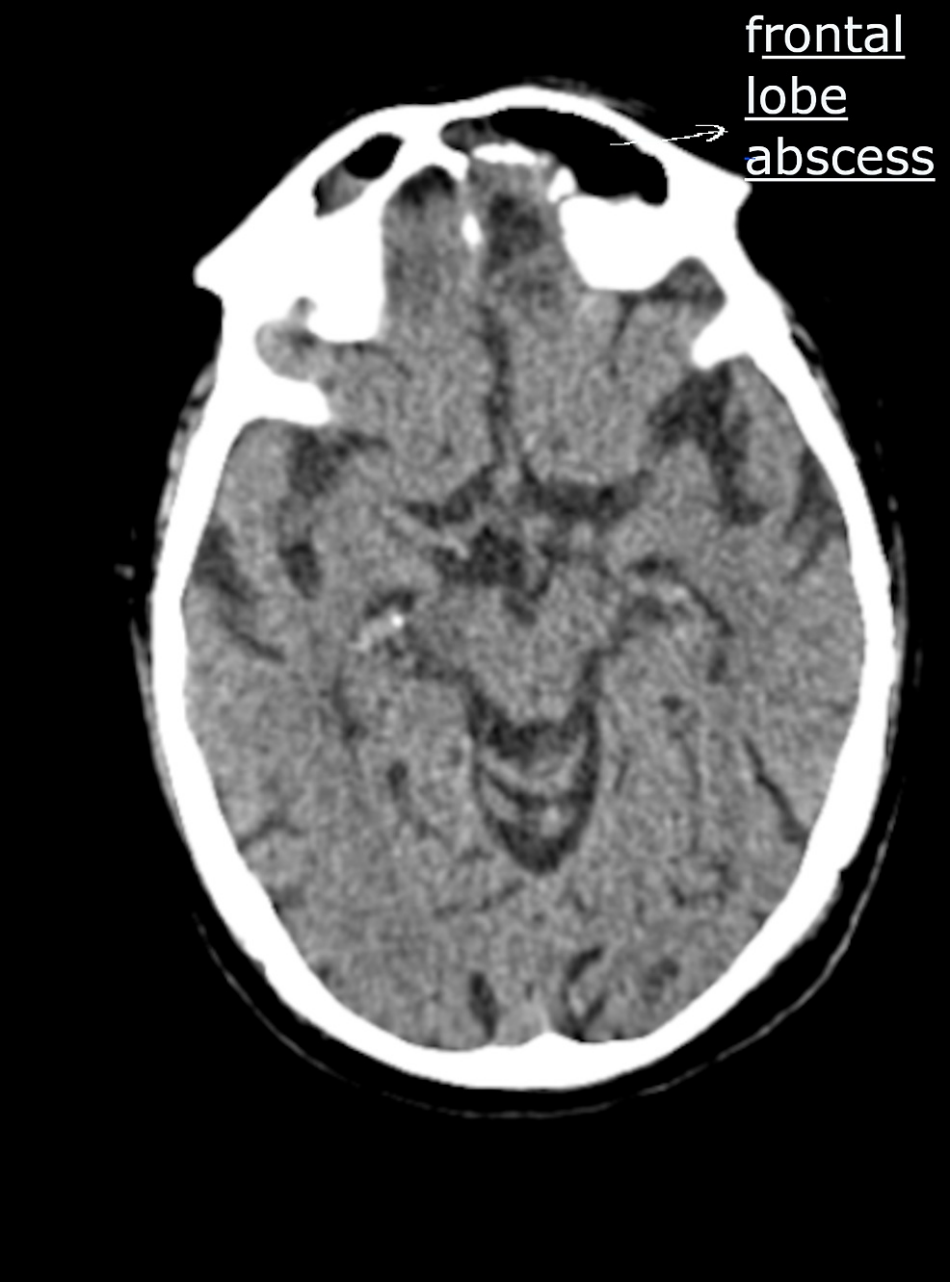
CT brain showing frontal lobe abscess

On examination, he was stuporous (GCS-8), afebrile to touch, pulse of 128 beats/min, regular, blood pressure of 138/94 mmHg and oxygen saturation of 94% on room air. He was administered antiepileptics and was immediately taken for a CT scan of the brain which revealed sino-orbital mucormycosis with abscess in the frontal lobe (Figure 1).

One month earlier, he was diagnosed with severe COVID-19 pneumonia and was promptly treated with steroids (low dose dexamethasone of 4mg/day for the duration of three days), Remdesivir and bilevel positive airway pressure (BiPAP) support (for two weeks) as per conventional guidelines. He is a diabetic diagnosed before 10 years and was taking oral antihyperglycemic medicines for the same.

The patient was admitted to medicine ICU. A neurosurgeon opinion was taken regarding the same. No feasible intervention was planned considering the age and extent of the spread of infection.

In the MICU, he was vitally stable with complete loss of vision. His blood investigations revealed renal and liver function tests were normal (Table 1).

**Table 1:** Blood investigations on presentation g- gram; dl- deciliter; cu.mm- cubic millimeter; fL- femtoliter; pg- picogram; mcg- microgram; MCV- mean corpuscular volume; MCH- mean corpuscular hemoglobin; MCHC- mean corpuscular hemoglobin concentration.

Investigation	Result
Hemoglobin	11 g/dl
Hematocrit	33.9%
RBC count	5.1 million/cu.mm
MCV	75.2 fL
MCH	22.7 pg/cell
MCHC	30.2 g/dL
Total Leukocyte count	18,000 cells/cu.mm
Differential counts: neutrophils	40%
Differential counts: lymphocytes	54%
Differential counts: monocytes	4%
Differential counts: eosinophils	2%
Platelet counts	2.08 X 10^5^ cells/cu.mm
Peripheral smear	Predominantly Normocytic severely hypochromic RBCs with moderate anisopoikilocytosis showing few microcytes and pencil cells.
Dimer	0.6 mcg/ml
HbA1c	6.1%

The patient was started on IV antibiotics, insulin, intra-orbital and systemic liposomal amphotericin-B injections at 10mg/kg/day and other symptomatic management. The patient underwent multiple functional endoscopic sinus surgeries (FESS) to look for resolution of the disease. After completion of 21 doses of amphotericin-B, the patient was discharged in stable condition.

## Discussion

Secondary infections in COVID-19 recovered patients are a common phenomenon. The most prevalent infections were respiratory, blood-stream, and urinary infections, with gram-negative bacteria (26, 50.00%) being the most commonly detected pathogen, followed by gram-positive bacteria (14, 26.92%), virus (6, 11.54%), fungi (4, 7.69%), and others (2, 3.85%). These infections can be caused by a complex interaction of variables, including underlying illnesses including diabetes mellitus, prior pulmonary pathology, immunosuppressive treatment, the risk of hospital-acquired infections, and systemic immunological changes caused by COVID-19 infection itself [2].

COVID-19 infection has a predisposition for developing severe lung disease and subsequent alveolo-interstitial pathology, which could enhance the risk of invasive fungal infections. Second, COVID-19's immunological dysregulation, which includes a decrease in T lymphocytes, CD4+ T cells, and CD8+ T cells, may have an impact on innate immunity [5].

In the presence of predisposing conditions such as those listed above, early diagnosis of mucormycosis is of great importance for timely treatment and better prognosis of the patient [7,8]. Treatment should be started as soon as possible to reduce mortality. It is necessary to use a multidisciplinary team approach.

Uraguchi et al. in their study had treated a case of rhinocerebral mucormycosis with brain abscess drained by endoscopic endonasal skull base surgery in a 70-year-old male [9].

Choir et al. described a 49-year-old man who developed left ophthalmoplegia and left hemiparesis due to an abscess in the right frontotemporal region for which they treated her with amphotericin-B injections and the patient was discharged in stable condition [10].

Mucormycosis is treated with a combination of surgical debridement and antifungal medication [11]. The treatment of choice is liposomal amphotericin-B at a starting dose of 5 mg/kg body weight (10 mg/kg body weight if central nervous system [CNS] involvement is present). It is incompatible with normal saline/Ringer Lactate and should be diluted in 5% or 10% dextrose.

It has to be continued till a favorable response is achieved and disease is stabilized which may take several weeks following which step down to oral posaconazole (300 mg delayed-release tablets twice a day for one day followed by 300 mg daily) or isavuconazole (200 mg one tablet three times daily for two days followed by 200 mg daily) can be done.

The therapy is to be given till there is a resolution of clinical features of infection and resolution of radiological features of active infection along with abatement of the risk factors like hyperglycemia, immunosuppression, steroid use, etc. [12]. It might have to be administered for an extended period of time. In case if conventional amphotericin-B (deoxycholate) is given, the dose is to be kept at 1-1.5mg/kg/day and renal functions and electrolytes must be normal.

The patient we described with severe COVID-19 was a long-standing diabetic. After one month of being admitted for moderate COVID-19 infection for which he was kept on non-invasive ventilation (NIV) for three days and getting discharged, he presented to the emergency department with status epilepticus. After MRI brain, the features of sino-orbital infection with cerebral mucormycosis and frontal lobe abscess were discovered, he was treated with antiepileptics, antifungals. All of these variables, as well as any potential COVID-19 pathophysiological processes, are likely to promote fungal co-infection.

There are many complications of COVID-19, out of which mucormycosis has gained prominence in recent times. Patients with mucormycosis in the post-COVID phase have various complications like sinusitis, orbital cellulitis, and intracranial extensions of the mucormycosis. This infection seen in the patient was probably a result of multiple contributing factors such as diabetes mellitus, use of corticosteroids, use of BiPAP support, and COVID-19 pneumonia in the young elderly. Diabetes is a known risk factor for mucormycosis for long due to hyperglycemia-induced release of free iron and up-regulation of GRP-78 receptor ultimately causing increased fungal binding and invasion as a result of increased expression of fungal spore coat protein homologs (CotH proteins) [13]. In this patient, the HbA1c values indicated a well-controlled diabetes for the last three months and the patient had received only low dose steroids for the duration of mere three days but still the patient contracted mucormycosis, which may be due to immunocompromised state with COVID-19 infection. COVID-19 in itself leads to immune dysregulation which has been reported before and the patient was also given BiPAP support which should have further facilitated mucormycosis in an already predisposed elderly male [14]. COVID-19 leads to downregulation of the complement system and defective phagocytosis. Also, there is disruption of the tight junctions of natural barriers of the immune system by SARS-CoV-2 [14]. Thus, mucormycosis as a complication of post-COVID sequelae due to COVID-19-induced immunosuppression can be a rare but important manifestation presenting as status epilepticus mimicking a seizure disorder.

## Conclusions

COVID-19 is linked to a high rate of secondary infections, both bacterial and fungal, most likely as a result of immunological dysregulation. Furthermore, the extensive use of steroids, monoclonal antibodies, and broad-spectrum antibiotics as part of the COVID-19 armamentarium may result in the development or aggravation of preexisting fungal infections. Physicians should be vigilant of the possibility of invasive secondary fungal infections in patients with COVID-19 infection, especially those with preexisting risk factors, and be able to diagnose and manage these infections early, minimizing mortality and morbidity. It is important for the physicians to note that even individuals with well-controlled blood glucose levels and not receiving high dose steroids may develop mucormycosis as a COVID-19 sequelae due to immune dysregulation.
